# Applications of Metabolomics to the Clinical Management of Breast Cancer: New Perspectives for Diagnosis, Treatment and Prognosis

**DOI:** 10.3390/ijms27052114

**Published:** 2026-02-24

**Authors:** Yuqiu Li, Hongnan Mo

**Affiliations:** Department of Medical Oncology, National Cancer Center/National Clinical Research Center for Cancer/Cancer Hospital, Chinese Academy of Medical Sciences and Peking Union Medical College, No. 17 Panjiayuan Nanli, Chaoyang District, Beijing 100021, China; liyuqiu@student.pumc.edu.cn

**Keywords:** breast cancer, metabolomics, precision medicine, metabolic features

## Abstract

Breast cancer is a heterogeneous malignancy that often changes during diagnosis and treatment, so timely monitoring of tumors, patients and treatment responses is crucial to improve the prognosis of patients. With the development of precision oncology, early patient stratification and the formulation of tailored therapeutic approaches have become essential strategies to maximize treatment efficacy. Several techniques, such as molecular pathology and genomics analysis have been thoroughly studied in the diagnosis and treatment of breast cancer, but they only evaluate and analyze from the perspective of patients or tumors in isolation. Metabolomics uses high-throughput analytical techniques to provide a functional readout of the biological phenotype, reflecting the sum of alterations occurring at the DNA, RNA, and protein levels. Therefore, through the detection of tumor tissues and peripheral blood of patients, metabolomics could describe the bidirectional interaction between the tumor and its microenvironment, as well as the systemic metabolic changes in patients to evaluate cancer progression from both tumor and patient aspects in a more comprehensive way. In this review, we summarize the currently available techniques for metabolomics and how metabolomics can be used to improve the clinical management of breast cancer patients, including diagnosis, treatment, and prognosis. We also discuss current challenges and future directions in metabolomics research.

## 1. Introduction

Breast cancer (BC) is the most common malignant tumor and the leading cause of mortality among women worldwide, with more than 2.2 million new cases in 2020 [1]. As a disease characterized by significant heterogeneity in its molecular profile and cellular composition, BC requires precise molecular subtyping to guide diagnosis, therapeutic strategies, and prognostic assessments [2,3,4,5]. Based on the tumor size, histological grade, immunohistochemistry of ER/PR status, and the amplification of HER2, BC tumors have been classified into four major intrinsic molecular subgroups [6]. Each BC subtype is defined by distinct biological characteristics, prognostic implications, and tailored treatment approaches [7,8,9,10]. However, these classifications still do not fully account for the diversity of clinical behavior, such as differences in treatment response and risk of recurrence [11]. In addition, the famous BRCA (Breast Cancer Susceptibility Gene) detection, the 21-gene recurrence score (Oncotype DX), and the 70-gene signature (MammaPrint) have been widely used in the screening of high recurrence populations to guide the postoperative adjuvant therapy of BC patients [12,13,14]. However, these genetic testing methods are only from the patient level, such as the germ line, or only from the tumor level to assess and predict and assess the development of BC. Therefore, more efforts are needed to refine the molecular classification framework to precisely classify the BC subtypes and comprehensively evaluate tumor progression from multiple perspectives.

Metabolomics uses high-throughput analytical techniques focused on the systematic identification and quantification of small-molecule metabolites (1500 Daltons or less and non-peptide) within biological specimens [15]. These metabolites, including lipids, amino acids, carbohydrates, and nucleotides, represent the downstream products of genomic, transcriptomic, and proteomic activity [16,17]. Metabolomics delivers a functional readout of metabolic processes and describes the sum of alterations occurring at the DNA, RNA, and protein level, thereby directly reflecting cellular physiology and often serving as the most sensitive method to detect pathological alterations [18]. Conversely, metabolites could regulate protein function, influencing nearly all biological processes from DNA replication to RNA transcription and translation [19]. Therefore, it can act as a dynamic biomarker, participating in diverse processes such as epigenetic regulation, tumorigenesis, cancer cell invasion, and other cellular processes. In addition, metabolomics elucidates hallmarks of malignancy, such as metabolic reprogramming and the alterations of tumor environment, which can enable therapeutic interventions that reshape the genome, transcriptome, and proteome [19,20].

The occurrence and development of BC are closely related to metabolic reprogramming, which is an important basis for BC to use metabolomics to improve clinical decision-making and promote the realization of precision medicine [21]. A series of studies have revealed that BC cells display significant metabolic reprogramming that enables them to survive and rapidly proliferate in a nutrient-poor tumor microenvironment [22]. For example, the metabolic reprogramming of glycolysis, oxidative phosphorylation, amino acid metabolism, lipid metabolism, and other branched pathways in triple-negative breast cancer (TNBC) fulfills bioenergetic and biosynthetic demands of tumor cells, maintains the redox balance, and further promotes oncogenic signaling, cell proliferation, and metastasis [23].

Currently, most reviews about BC metabolomics mainly focus on diagnostic biomarkers or fundamental biochemical pathways of metabolic reprogramming. However, a comprehensive summary regarding the application of metabolomics in defining BC subtypes and predicting recurrence, metastasis, and treatment response remains missing. In our review, we highlight how metabolomics can be used not only for diagnosis but also for monitoring recurrence, predicting treatment efficacy and drug resistance, and evaluating long-term prognosis. Our review is, to our knowledge, the first to focus on the role of metabolomics across the entire clinical management of BC. By providing this integrated perspective, we aim to offer a practical roadmap for implementing metabolomics in precision oncology. Furthermore, in today’s era of rapid development of omics, we also summarized the problems faced by metabolomics and the future development direction, which is crucial for facilitating the transformation of metabolomics from the laboratory stage to truly clinical practice.

## 2. Metabolomics Approaches in Cancer Research

### 2.1. Methodology and Instrumentation

Nuclear magnetic resonance (NMR) spectroscopy and mass spectrometry (MS), such as liquid chromatography-MS (LC-MS) and gas chromatography-MS (GC-MS), are mainly analytical techniques [24,25]. As the most widely used analytical technique in metabolomics, MS offers high sensitivity and resolution for detecting diverse metabolites. GC-MS offers high sensitivity but typically requires chemical derivatization to enhance metabolite volatility, limiting its utility for non-volatile or phosphorus-containing compounds such as lipids and nucleotides [26]. In contrast, LC-MS avoids derivatization and is versatile for analyzing biofluids and polar/nonpolar metabolites, including challenging phosphorus-rich molecules [26]. However, LC-MS needs tailored chromatographic columns and mobile phases for distinct compound classes. LC-MS remains the most widely adopted method due to its broad metabolite coverage, compatibility with complex biological matrices, and high sensitivity [18]. NMR spectroscopy, though less sensitive than MS, provides non-destructive, reproducible quantification of metabolites and is particularly effective for studying biofluids such as serum and urine [27]. Both NMR and MS techniques can be used in targeted and untargeted analytical approaches.

Targeted metabolomics focuses on the quantitative or semi-quantitative analysis of a limited number of metabolites (a few hundred), enabling precise concentration comparisons across datasets [28]. This approach is ideal for hypothesis-driven studies but captures only a fraction of the metabolome. It offers high sensitivity for specific low-concentration metabolites but lacks breadth [29]. In contrast, untargeted metabolomics aims to detect all measurable metabolites (thousands of features), generating large-scale, discovery-oriented data. Untargeted metabolomics excels at discovering new biomarkers, but faces challenges such as difficulties in metabolite annotation, insufficient sensitivity of low abundance molecules, and poor cross platform data comparability [30]. Consequently, integrating both approaches that use untargeted metabolomics to discover novel biomarkers and using targeted metabolomics to quantitatively validate metabolomes are increasingly being adopted to maximize metabolome coverage and translational relevance [25,31].

### 2.2. Source Material

Metabolomics can be performed on a diverse array of biological specimens, such as clinical materials include plasma, serum, urine, and surgical tissue extracts or tumor biopsies, which provide direct insights into disease-associated metabolic alterations. A series of studies also revealed that non-conventional biofluids such as cerebrospinal fluid, saliva, sputum, breast milk, and fecal water, as well as amniotic fluid, seminal plasma and bronchial washings [32,33,34,35,36]. In addition, cells and tissues cultured in the laboratory, specimens collected from laboratory animals, could also be used in the metabolomics analysis [37,38]. The metabolomics of blood samples can be more comprehensive, showing the metabolic status influenced by tumor, patients, intestinal microbes, treatment and other aspects, and can be dynamically monitored for easy adoption. The metabolomics of tumor biopsy tissue can directly reflect the internal metabolic state of the tumor, and can be correlated with the genome of tumor cells and the characteristic spectrum of immune cells, which is helpful to explain the cause of immune cold tumor of BC.

In BC research, the plasma, serum and tumor biopsies of BC patients are the most predominant specimens. In addition, the BC cells cultured in laboratories and tumors of mice are also commonly used.

## 3. Applications of Metabolomics in the Clinical Management of Breast Cancer

**Figure 1:**
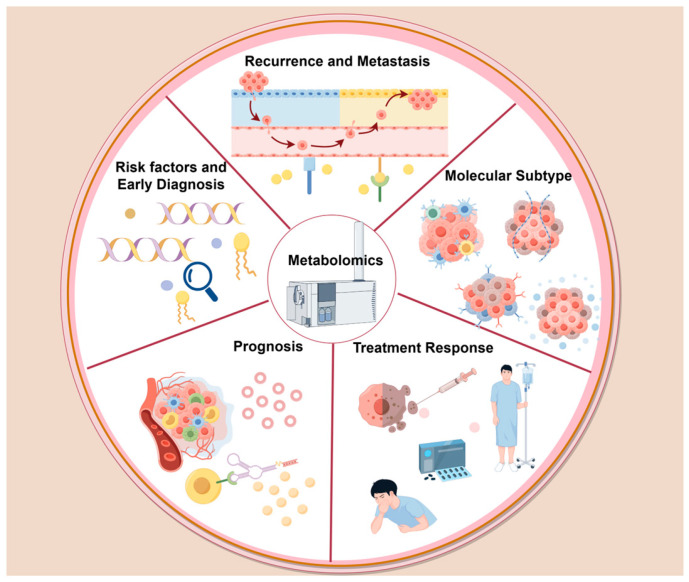
The application of metabolomics in BC research. Metabolomics promotes the discovery of biomarkers and related mechanisms to the diagnosis, molecular subtyping, metastasis and recurrence, chemoresistance, treatment-related adverse events and prognosis of BC. The figure was created by Figdraw (https://www.figdraw.com/ (accessed on 4 January 2026)).

Metabolomics plays an important role in the diagnosis, molecular subtyping, metastasis and recurrence, chemoresistance, treatment-related adverse events and prognosis of BC (Figure 1).

### 3.1. Identifying Breast Cancer Risk Factors and Biomarkers for Early Diagnosis

Multiple metabolomics studies have found several metabolites are associated with an increased risk of BC, leading to new insights for the etiology and prevention of BC (Table 1). In a nested case–control study within the American Nurses’ Health Study (NHS), researchers used plasma metabolomics via liquid chromatography tandem mass spectrometry to analyze the plasma samples collected at 10 years and <10 years before diagnosing BC [39]. Phenylalanine, proline, and triacylglycerols (TAG) with less than three double bonds had positive associations with BC risk, while cholesteryl esters and TAG with more than three double bonds were inversely related with risk [39]. However, these metabolites only stood out as nominally significant, and no individual metabolites were found to have associations with BC risk after correlation for several effective tests [39]. In the French E3N cohort, NMR-based untargeted metabolomics revealed that 10 metabolites were associated with BC risk in the premenopausal subgroup, especially histidine, N-acetyl glycoproteins, glycerol and ethanol [40]. Similar conclusions were also found in Wu et al.’s research, which indicated that amino acid and lipid metabolism may be associated with BC risk [41].

As for BC diagnosis, the early screening of BC mainly relies on traditional imaging methods such as mammography, but it also has some limitations, including insufficient sensitivity to dense breast tissue, a high false positive rate, and difficulty in detecting small nodules or carcinoma in situ [42]. In addition, the risk of radiation exposure and discomfort during the examination further limit its widespread use. In order to break through these bottlenecks, metabolomics technology has gradually become a research hotspot because of its high sensitivity and functional information mining ability. Combining untargeted metabolomics and lipidomics, Nguyen Ky Anh et al. found that ethers linked to phosphatidylcholine were significantly different between invasive catheter carcinoma and benign tumors, including cases with inconsistent mammogram results. This indicated the potential of metabolic biomarkers to complement other criteria in BC screening [43]. In addition, there were significant changes in multiple lipids such as sphingomyelin, triacylglycerol, and free fatty acid and hydrophilic metabolites including glutamate, glycochenodeoxycholate, and dimethyluric acid in the BC group [43]. Wei et al., combining non-targeted liquid chromatography–Quadrupole time-of-flight mass spectrometry (LC-QTOF-MS) and targeted LC-QQQ-MS data, identified 33 differential metabolite/mass spectrum features [44]. Among them, ethyl (R) -3-hydroxycaproate, capylic acid, hypoxanthine and three unannotated characteristics (*m*/*z* 358.0018, 354.0053, 356.0037) were identified as key markers of detecting early BC [44]. Combined analysis further revealed eight metabolic pathway abnormalities associated with early BC, including fatty acid biosynthesis, fatty acid and aminoacyl-tRNA biosynthesis, and inositol phosphate metabolism [44]. This study not only demonstrates the high precision advantage of multi-platform metabolomics integration in the diagnosis of early BC, but also sheds light on the underlying pathological mechanisms of early BC, providing a new perspective for improving prognosis. Another research showed that the four-metabolite combination consisting of N-acetyl-D-tryptophan, 2-arachidonoylglycerol, pipecolic acid and oxoglutaric acid screened by metabolomics had significant diagnostic efficiency of BC [45]. By comprehensive analyzing the metabolomics and transcriptomics data, it provides an important theoretical basis for developing new diagnostic markers and metabolic intervention strategies in TNBC. Although these studies demonstrate the power of metabolomics in the diagnosis of BC, highlighting its potential as a complementary imaging tool, the clinical translation of metabolic markers still requires large-scale cohort validation and the development of standardized testing procedures.

Metabolomics could help reveal the heterogeneity of BC and identify marker differential metabolites between subtypes, which provides new targets for non-invasive diagnosis, and also lays a theoretical basis for the development of metabolic intervention therapy. The combination of N-acetyl-D-tryptophan and 2-arachidonoylglycerol can also effectively distinguish TNBC from non-TNBC [45]. For TNBC, the literature revealed that 7-methylguanine could be considered as a potential biomarker in diagnosing TNBC based on LC-MS [46]. Furthermore, they also found that the disturbance of energy metabolism in TNBC is closely related to tyrosine metabolism, phenylalanine metabolism and the glycolysis/gluconeogenesis pathway. Key metabolites such as 4-hydroxyphenylglyoxal and oxaloacetic acid, and regulatory genes such as PCK1 and MAOA, were considered as vital components [46]. Yang et al. [47] integrated PET/MR imaging, transcriptomics, and NMR-based metabolomics to reveal distinct metabolic profiles between luminal A and luminal B BC subtypes. The study highlighted elevated glucose metabolism in tumor centers and enhanced lipid and one-carbon metabolism in peripheral regions, particularly in luminal B tumors, which may be used as an early diagnostic marker of luminal B subtype [47]. Key metabolites such as acetate-, serine-, and choline-related compounds, along with lipid metabolism genes, were identified as potential biomarkers for early detection and subtype differentiation of luminal A and luminal B subtypes [47].

**Table 1:** Summary of metabolomics biomarkers for breast cancer diagnosis.

Sample Type	Technique Used	Changes in Metabolites	Reference
Circulating plasma from BC patients	NMR-based untargeted metabolomics	Histidine (↑), Glycerol (↑), NAC (↑), Ethanol (↑)	[40]
Plasma from BC patients	HILIC with LC-HRMS	1,3-Dibutyl-1-nitrosourea (↑), L-Histidine (↓), N-(6)-Methyllysine (↑), N-Acetylgalactosamine (↓), 11-cis-Eicosenoic acid (↑), LysoPE(0:0/24:6(6Z,9Z,12Z,15Z,18Z,21Z) (↑)	[41]
Breast tumor tissues	LC-MS/MS	Glutamate (↑), Glycochenodeoxycholate (↑), Ether-linked phosphatidylcholine (↑), Sphingomyelin (↑), Triacylglycerol (↑), Free fatty acids (↑), Dimethyluric acid (↑)	[43]
Plasma from BC patients	LC-MS	Ethyl (R)-3-hydroxyhexanoate (↑), Caprylic acid (↓), Hypoxanthine (↑), *m*/*z* 358.0018(↓), 354.0053 (↓), 356.0037 (↓)	[44]
Serum from BC patients	UHPLC-MS-based untargeted metabolomics	BC: N-acetyl-D-tryptophan (↑), 2-Arachidonoylglycerol (↑), Pipecolic acid (↑), and Oxoglutaric acid (↑) TNBC: N-acetyl-D-tryptophan and 2-Arachidonoylglycerol	[45]
Serum and plasma from TNBC patients	UPLC-QTOF/MS	7-Methylguanine (↑), Taurine (↑), Hypotaurine (↑), Glycerol-3-phosphate (↓), Succinate (↓), Choline (↓), Serine (↓), Glycine (↓), Alanine (↓)	[46]
Central and peripheral BC tissues	NMR spectroscopy	Acetate (↑ in luminal B compared to luminal A), Serine (↑ in luminal B compared to luminal A), Choline, Phospholipid metabolism products (↓ in central compared to peripheral), TCA cycle intermediates (↑ in central compared to peripheral)	[47]

↑—increase, ↓—decrease.

### 3.2. Anticipating Diagnosis of Recurrence and Metastasis

Although the early diagnosis and treatment of BC have significantly improved the prognosis of patients, recurrence and metastasis are still major clinical challenges [48]. Traditional imaging, such as chest computed tomography for lung metastases, liver ultrasound for liver metastases, and bone scans for bone metastases, and tissue biopsy have the limitations of insufficient sensitivity and high invasiveness in the early diagnosis of metastasis. Although the development of metabolomics cannot replace the gold standard of diagnosis provided by imaging and pathology, its high sensitivity may potentially facilitate the early detection of recurrence and metastasis [49,50,51].

Recent studies have demonstrated that metabolomics is a powerful tool for identifying biomarkers of BC recurrence and metastasis (Table 2). Regarding recurrence, Yang et al. [52] identified plasma valine level as a promising predictor of BC recurrence, with higher levels associated with reduced risk, particularly in ER-negative and PR-negative subgroups. Other metabolites related to glycine, serine, and branched-chain amino acid metabolism were also linked to recurrence. The integration of metabolomics into multi-modal machine learning models has significantly improved the prediction of overall recurrence risk. In the HR+/HER2- subtype, researchers developed the CIMPTGV model based on clinical information, immunohistochemistry, metabolomics, pathomics, transcriptomics, genomics, and copy number variations to predict recurrence risk [53]. Metabolomics revealed that high-risk recurrence groups exhibit a significant enrichment of nucleic acid metabolites, such as pseudouridine and N4-acetylcytidine, which are closely linked to genomic instability and high homologous recombination deficiency (HRD) scores. A marked reduction in lipids, including eicosadienoic acid and linoleic acid, serves as a metabolic signature for poor prognosis [53]. For metastasis, by integrating plasma proteomics and metabolomics techniques, Ye et al. [54] identified distinct biomarker panels for each BC metastasis type: ECM-related proteins and metabolites such as leucyl-tryptophan, LysoPC(P16:0/0:0), FN1, and HSPG2 for bone metastases, retinol metabolism-linked metabolites such as dUDP, LPE(18:1/0:0), and aspartylphenylalanine for liver metastases, and inflammation-associated metabolites such as testosterone sulfate and PE(14:0/20:5) for lung metastases. Notably, metabolomics outperformed proteomics in distinguishing liver and lung metastases, highlighting its sensitivity to microenvironmental dynamics [54]. However, a major limitation is the small sample size in current studies, and the transformation of metabolic profiles during metastasis such as the transition from glycolytic to oxidative phosphorylation complicates the identification of static biomarkers. Future integration with spatial or single-cell approaches could further unravel metastatic heterogeneity, advancing precision oncology strategies.

Beyond biomarker discovery, metabolomics also offers insights into the mechanisms of BC metastasis and recurrence. For instance, NMR-based metabolomic analysis has revealed that RON-DEK-β-catenin signaling axis drives metabolic plasticity of BC cells to support metastasis by regulating pathways such as glycolysis, TCA cycle, NAD+ metabolism and creatine kinetics [55]. By integrating these metabolic changes with gene expression data, researchers have developed novel metabolic signatures that significantly improve survival risk stratification and the prediction of chemotherapy response [55]. Furthermore, hypoxanthine has been identified as a novel driver of metastasis by metabolomics [56]. Secreted by highly metastatic BC cells, it induced epithelial–mesenchymal transformation (EMT), promoted angiogenesis via vascular endothelial growth factor (VEGF) and platelet-derived growth factor (PDGF) signaling, and regulated lipid metabolism [56]. Collectively, these findings underscore the potential of metabolomic analysis to identify key biomarkers and therapeutic targets, providing a theoretical basis for multi-omics-based tools in predicting BC recurrence and metastasis.

**Table 2:** Summary of metabolomics biomarkers for breast cancer recurrence and metastasis.

Sample Type	Technique Used	Changes in Metabolites	Reference
Plasma from recurrent BC patients	GC-MS LC-MS UHPLC-QTOF-MS	Valine, creatine, methionine	[52]
Plasma from recurrent BC patients	LC-MS/MS	Pseudouridine, N4-acetylcytidine, and 5′-methylthioadenosine, eicosadienoic acid, linoleic acid	[53]
Plasma from metastatic BC patients	UPLC-MS	Bone metastasis: leucyl-tryptophan, LysoPC(P-16:0/0:0), fibronectin 1 and heparan sulfate proteoglycan 2 Liver metastasis: LPE(18:1/0:0), dUDP, aspartylphenylalanine Lung metastasis:dUDP, testosterone sulfate, PE (14:0/20:5)	[54]
Conditioned media from MDA-MB-231 and MCF-7 cells	H-NMR	Hypoxanthine (in highly metastatic MDA-MB-231 compared to less metastatic MCF-7)	[56]

### 3.3. Refining Breast Cancer Molecular Subtypes Based on Metabolic Profile

Through the analysis of the genome, transcriptome, proteome, metabolomics, and imaging pathological features, the integration of multi-omics analysis promotes the innovation of BC molecular typing and treatment strategies towards dynamic and racial differentiation. One of the most mature and successful cases is the molecular subtyping of TNBC.

**Figure 2:**
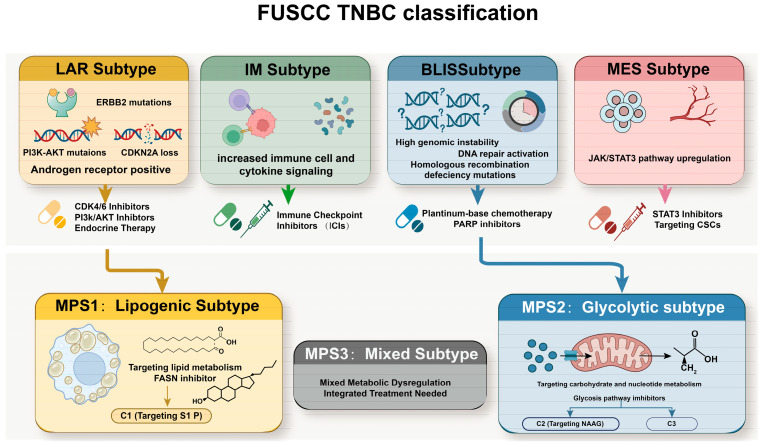
Schematic integration of FUSCC molecular subtyping of TNBC. TNBCs are classified as four major subtypes: luminal androgen receptor (LAR), immunomodulatory (IM), basal-like immune-suppressed (BLIS), and mesenchymal (MES) subtype. Each subtype is characterized by distinct genomic alterations, signaling pathway activation, and potential therapeutic targets. Additionally, three metabolic pathway subtypes are highlighted as MPS1, MPS2, and MPS3 subtypes. The LAR subtype almost corresponds to the MPS1, which is further stratified as the metabolomic C1 subtype. The BLIS subtype almost aligns with MPS2 and encompasses the metabolomic C2 and C3 subtypes.

Currently, the most popular subtyping of TNBC is the Fudan University Shanghai Cancer Center (FUSCC) TNBC classification (Figure 2). In 2019, based on the multi-omics data of 465 cases of TNBC in the Chinese population, including whole-exome sequencing, copy number variation, and RNA sequencing, Jiang et al. divided TNBC into four molecular subtypes: luminal androgen receptor (LAR), immunomodulatory (IM), mesenchymal (MES) and basal-like immunosuppressed (BLIS) [57]. Each subtype presents distinct characteristics and therapeutic vulnerabilities. The LAR subtype is an androgen receptor (AR)-positive TNBC that shows dependence on androgen receptors [58]. While AR expression often correlates with a favorable prognosis, the survival benefit of anti-androgen therapy remains controversial [59,60,61,62,63]. LAR subtypes are often linked to a more frequent occurrence of phosphatidylinositol 3-kinase catalytic alpha (PIK3CA) mutations and ERBB2 mutations and a higher frequency of CDKN2A loss, indicating a potential benefit of targeting ERBB2, PI3K, and AKT inhibitors and CDK4/6 inhibitors [57]. Each subtype presents distinct characteristics and therapeutic vulnerabilities. The LAR subtype is an androgen receptor (AR)-positive TNBC that shows dependence on androgen receptors [58]. While AR expression often correlates with a favorable prognosis, the survival benefit of anti-androgen therapy remains controversial [59,60,61,62,63]. LAR subtypes are often linked to a more frequent occurrence of PIK3CA mutations and ERBB2 mutations and a higher frequency of CDKN2A loss, indicating a potential benefit of targeting ERBB2, PI3K and AKT inhibitors and CDK4/6 inhibitors [57]. The IM subtype is characterized by immune activation and high immune cell infiltration. Due to its unique immune microenvironment and low mutation load, it is suitable for immune checkpoint inhibitors (ICIs). The MES subtype exhibits stem-cell properties and high plasticity, often leading to increased aggressiveness and metastasis. Therapeutic targets include the JAK/STAT3 pathway, angiogenesis, and cancer stem cells. [57]. Different from the IM subtype, the BLIS subtype has high cell-cycle gene expression and DNA repair deficiency. Patients with HRD in this subtype typically benefit from platinum-based chemotherapy or PARP inhibitors [57].

**Figure 3:**
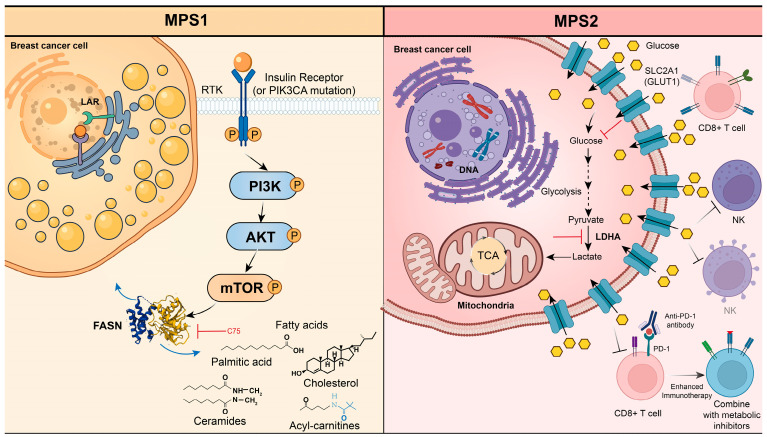
Molecular and metabolic features of the MPS1and MPS2 subtypes. MPS1 cell lines, characterized by frequent mutations in the PI3K and RTK-RAS pathways, are sensitive to inhibitors of lipid synthesis such as C75. Activation of the PI3K pathway could drive nutrient uptake and enhance lipid synthesis in tumor cells. In the MPS2 subtype, metabolic reprogramming is driven by genomic alterations that enhance glycolysis. Key therapeutic targets include the glucose transporter SLC2A1 and LDHA, whose inhibition disrupts glycolytic flux and promotes cancer cell apoptosis. Furthermore, LDHA blockade enhances anti-tumor immunity by increasing CD8^+^ T and NK cell infiltration, thereby sensitizing tumors to anti-PD-1 immunotherapy.

The emergence of metabolomics has gradually become an indispensable part of driving the construction of a multi-omics subtype system by systematically analyzing the dynamic reprogramming of lipid, amino acid and energy metabolic network in BC. In the FUSCC study, TNBC is classified into three metabolic-pathway-based subtypes (MPSs) through metabolite pathway enrichment analysis (MPEA) [64]. Firstly, MPS1 stands for the lipogenic subtype (26.4%). IT mainly contains the LAR subtype and the PAM50 non-basal subtype. The subtype exhibits relatively enhanced fatty acid and cholesterol synthesis, such as myristic acid, palmitoleic acid, and arachidonic acid, driven by high-frequency PI3K and RTK-RAS pathway mutations. MPS1 tumors often occur in elderly patients and, despite higher axillary lymph node positivity, show a favorable prognosis. These tumors are sensitive to lipid synthesis inhibitors like C75. Secondly, MPS2 is the glycolytic subtype (36.9%) with upregulated carbohydrate and nucleotide metabolism, including the citric acid cycle, glycolysis, purine metabolism, and pyrimidine metabolism. Most MPS2 cases belong to the BLIS subtype and are characterized by high chromosomal instability. The study suggests that solute carrier family two facilitated glucose transporter member 1 (SLC2A1) and lactate dehydrogenase A (LDHA) may be potential drug targets for cancer treatment. Inhibition of SLC2A1 or LDHA affects the glycolytic pathway of cancer cells, ultimately leading to apoptosis [65]. In addition, inhibition of LDHA could increase the tumor-infiltrating CD8 + T and NK cells in the mice model, therefore enhancing tumor response to anti-PD-1 immunotherapy in the MPS2 subtype [66] (Figure 3). MPS2 had a higher tumor grade and significantly worse RFS. Thirdly, MPS3, the mixed subtype with combined dysregulation of metabolic pathways, may require a more integrated treatment approach to address its complex metabolic profile.

Moreover, to further reveal the metabolic heterogeneity of TNBC, FUSCC conducted a comprehensive polar metabolomic and lipomic analysis of 330 TNBC samples and 149 paired normal breast tissues in 2022, which established the first metabolomic map of TNBC [67]. Based on metabolite characteristics, TNBC was divided into three distinct subtypes (C1–C3), and several potential therapeutic targets for each subtype were identified. C1 subtypes are characterized by high levels of ceramides and fatty acids. Characterized by high levels of sphingolipids and fatty acids, this subtype overlaps with the LAR transcriptomic subtype. The C2 subtype, enriched with oxidation and glycosylation metabolites, exhibited the upregulation of oxidation reactions and metabolites associated with glycosylation transfer, such as oxidized glutathione GSSG and uridine diphosphate glucose UDPG. Targeting N-acetylaspartyl-glutamate (NAAG) biosynthesis may be a viable therapeutic strategy. The C3 subtypes exhibit metabolic disorders, with smaller metabolic abnormalities and a lower likelihood of recurrence compared to healthy tissue. The BLIS subtype includes the C2 and C3 subtypes of metabolomics [67]. Further single-cell transcriptomic integration by Yu et al. categorized BC into two prognostic clusters based on four core metabolic pathways, including glycolysis, pentose phosphate pathway (PPP), fatty acid oxidation (FAO) and glutaminolysis [68]. Cluster 1 is characterized by high glycolytic activity, poor prognosis, and significantly shortened median survival. Cluster 2 is rich in fatty acid oxidation and glutamine decomposition and has significant survival advantages. They also found that malignant cells are the main drivers of metabolic heterogeneity, and their metabolic characteristics evolve dynamically during metastasis, such as the transformation of cluster 1 to cluster 2 in lymph node metastasis [68].

Metabolomics also reveals significant ethnic variations in BC. Analysis of the Chinese Breast Cancer Genome Atlas (CBCGA) cohort highlighted unique biological characteristics in the Chinese population [69]. They have found that AKT1 mutations are more common in the Chinese population, especially in the luminal A subtype, compared with the white BC population. High-frequency AKT1 mutations in Asian populations are significantly associated with glutamine metabolic dependence mediated by the PI3K/AKT/mTOR pathway, suggesting that metabolomics can reveal race-specific targets. In addition, Chinese patients have a higher proportion of HER2-enriched subtypes, especially in the HR+HER2+ subtype, suggesting that anti-HER2 therapy has a potential therapeutic effect in these patients. Sphingolipid metabolic disorders, such as ceramide metabolic imbalance unique to HER2-positive subtypes, form a positive feedback loop with ERBB2 gene amplification, which provides a theoretical basis for anti-HER2 therapy combined with sphingolipid pathway modulators [69]. Furthermore, studies also showed dUMP, L-octanoylcarnitine, L-proline, lysoPC(22:1), PS(22:0/0:0), and uric acid were highly correlated with five-year survival in TNBC patients [70].

Currently, the systematic metabolic classification for luminal and HER2+ types of BC is not as mature as that for TNBC, representing a critical area for future investigation to achieve truly comprehensive and precise treatment.

### 3.4. Evaluating Treatment Response in Breast Cancer Patients

#### 3.4.1. Identifying Biomarkers to Predict Treatment Response

Metabolomics provides a platform for predicting BC treatment response by dynamically monitoring tumor microenvironment and systemic metabolite changes (Table 3).

For neoadjuvant chemotherapy (NACT), baseline levels of specific metabolites can effectively predict pathological complete response (pCR). For instance, researchers have revealed that higher baseline levels of docosahexaenoic acid (DHA), preoperative glycine deoxycholic acid (GDCA), and glycine hyodeoxycholic acid (GHCA) were significantly associated with a higher likelihood of achieving pCR [71]. Conversely, elevated baseline levels of 3-indole sulfate, creatine, and uric acid are often observed in non-pCR groups [72]. The dynamic fluctuations of metabolites during treatment further reflect therapeutic sensitivity. Significant changes in asparagine, creatinine, and polyamine metabolites such as choline and carnitine during treatment suggest chemotherapy-induced metabolic reprogramming [72].

Metabolic biomarkers also demonstrate high specificity across different molecular subtypes. In the luminal type, therapeutic response is closely associated with negative correlations of ADP and hydroxyproline, whereas N1-acetylspermine serves as a positive predictor for pCR [72]. In TNBC type, unique metabolic profiles involving secondary bile acids and specific organic acids such as piperidinic acid play critical roles in predicting chemotherapy response [72]. In the HER2+ type, 3-indole sulfate was increased in the non-PCR group [72]. For patients with HER2 + BC treated with the TCbHP regimen (taxane, carboplatin, trastuzumab, and pertuzumab) during NACT, biomarkers including sophorobiose, n-(2-acetamido) iminodiacetic acid, taurine, and 6-hydroxy-2-aminocaproic acid, could be used to effectively distinguish TCbHP resistance from sensitivity, allowing for the early prediction of the TCbHP response to NAT in HER2 + BC patients [73]. Similarly, Miolo et al. [74] identified elevated serum levels of spermidine and tryptophan in patients achieving pCR in HER2 + BC patients undergoing NACT with trastuzumab-paclitaxel. These findings provide valuable insights into individual metabolic responses to therapy and may aid in identifying patients most likely to benefit from trastuzumab-paclitaxel treatment. Wei et al. [75] conducted a comparative serum metabolic profiling study of HER2 + BC patients who experienced pCR, partial response, or stable disease following NACT with epirubicin and cyclophosphamide, followed by doxorubicin in combination with trastuzumab. The study identified a progressive increase in threonine, glutamine, and linoleic acid levels in patients achieving pCR, with a subsequent decrease in isoleucine observed across all groups [75]. The above literature validated the potential of metabolomics for early prediction of treatment response and subtype stratification. However, more future studies are needed to validate these findings in larger patient populations and further explore their potential for clinical application.

#### 3.4.2. Revealing Mechanisms of Drug Resistance in Breast Cancer Treatment

Additionally, alterations in cellular metabolism have been implicated in the resistance to endocrine therapy, with evidence suggesting that changes in the metabolism of amino acids or glucose, as well as increased lipid levels, may play a role in the development of endocrine resistance in ER+ BC cells [76,77]. Furthermore, studies have suggested that metabolic reprogramming may also contribute to BC chemoresistance. Ahn et al. investigated the role of metabolic reprogramming, specifically enhanced mitochondrial fatty acid β-oxidation (FAO), in driving endocrine therapy resistance in ER+ BC [78]. The study identified that endocrine therapies like tamoxifen or fulvestrant activate FAO and oxidative phosphorylation (OXPHOS), which, in turn, promote resistance by activating the Src oncogenic pathway [78]. Using transcriptomics, metabolomics, and lipidomics in TNBC cells with carnitine palmitoyl transferase 1 (CPT1) inhibition, the authors derived a metabolic gene signature that was strongly associated with endocrine resistance in ER+ BC patients, linking FAO to poor clinical outcomes [78]. Targeting FAO or Src pathways, alone or in combination, represented a promising strategy to overcome resistance, supported by preclinical efficacy of FDA-approved inhibitors like etomoxir and dasatinib [78].

Current treatment of TNBC often involves the use of high-dose chemotherapy, which can cause severe side effects and lead to drug resistance [79]. There is an urgent need to find ways to reduce the dosage of chemotherapy in TNBC while maintaining or enhancing its effectiveness [80]. Researchers have shown that certain dietary compounds, such as polyphenols and omega-3 polyunsaturated fatty acids (PUFAs), can enhance the effectiveness of chemotherapy drugs like doxorubicin and overcome drug resistance in TNBC [81]. Through the use of untargeted metabolomics, researchers have identified a wide range of metabolites and metabolic pathways that are affected by these dietary compounds in MDA-MB-231 cells [81]. They revealed that these compounds do not uniformly target the same metabolic processes; instead, they clustered into distinct groups based on the similarities in the metabolic targets they affected. Key areas of metabolic impact include amino acid metabolism, with a particular focus on one-carbon and glutamine pathways, and changes in fatty acid oxidation [81]. They also observed that doxorubicin treatment on its own tends to target different metabolites and pathways compared to the chemosensitizing compounds [81]. These findings highlighted metabolomics-driven insights into multi-targeted chemosensitization mechanisms, proposing metabolic signatures including acylcarnitines and glutamine depletion as biomarkers for optimizing combination therapies [81]. Related to adriamycin, Ryu et al. discovered a correlation between glycolysis, lactate and ATP production, and resistance to adriamycin in MCF-7 cells. Their findings implied that modulating sulfur amino acid metabolism could be a potential therapeutic strategy for overcoming chemoresistance in cancer cells [82]. Cao et al. also utilized the MCF-7 cell line, noting that adriamycin slowed down various metabolic pathways, such as purine, pyrimidine, glutathione, and glycolysis, while also exacerbating oxidative stress [83]. In another study, Morvan observed that ascididemine exposure in MCF-7 cells led to an elevation in citrate, gluconate, and polyunsaturated fatty acids, along with a reduction in glycerophosphocholine and ethanolamine, indicative of heightened oxidative stress in vitro. This suggested that the central metabolic shifts in BC cells are a response to elevated oxidative stress [84].

Considering paclitaxel resistance, Kordias et al. investigated the metabolic mechanisms underlying chemoresistance in TNBC through multi-omics analysis of paclitaxel-resistant SUM159 cells (PTX-res) [85]. Transcriptomics revealed upregulation of cholesterol biosynthesis genes and ATP-binding cassette (ABC) transporters, while metabolomics and lipidomics identified significant alterations, including reduced myo-inositol levels, elevated free cholesterol, and increased phospholipid saturation [85]. Functional validation demonstrated that MSMO1 knockdown sensitized resistant cells to paclitaxel, linking cholesterol metabolism to chemoresistance. Integrated analysis highlighted metabolic vulnerabilities, particularly cholesterol biosynthesis and myo-inositol depletion, as potential therapeutic targets [85]. These findings underscored the role of metabolic reprogramming in paclitaxel resistance and propose MSMO1 inhibition or metabolic modulation as strategies to overcome chemoresistance in TNBC [85]. In a comparative analysis of metabolic pathways between luminal A BC cells and TNBC cells under paclitaxel treatment, Stewart et al. [86] identified distinct metabolic responses. Notably, choline and its metabolites were elevated in both luminal A and TNBC cell lines in the presence of paclitaxel. Intriguingly, choline, acetylcholine, phosphocholine, and sn-glycero-3-phosphocholine levels increased specifically in MDA-MB-468 cells under treatment, with the exception of sn-glycero-3-phosphocholine. Additionally, myo-inositol levels rose during treatment, being more pronounced in luminal A cells compared to TNBC cells. These observations highlighted the deregulation of glycolysis and glutathione pathways in cells treated with adriamycin and docetaxel, underscoring the need for further research into these biochemical pathways to enhance our comprehension of chemotherapeutic effects and potential resistance mechanisms [86].

#### 3.4.3. Predicting Treatment-Related Adverse Events

Treatment-related adverse effects such as neurotoxicity, metabolic disorders and cardiotoxicity remain important challenges in the treatment of BC. In recent years, metabolomics has provided a new perspective for predicting and monitoring adverse reactions of BC treatment through systematic analysis of small molecule metabolites in biological fluids. Piffoux et al. [87] demonstrated that untargeted high-resolution metabolomic profiling, combined with machine learning, effectively predicts long-term neurologic and metabolic toxicities in ER+/HER2- BC patients. Analyzing baseline serum samples from 992 participants in the prospective CANTO cohort, the research revealed that incorporating non-annotated and low-frequency metabolites significantly improved predictive accuracy compared to clinical variables alone, particularly for toxicity trajectories [87]. Key findings highlighted the role of exogenous metabolites (30–37% of predictive features) linked to environmental exposures, microbiota, and diet, alongside pathways such as amino acid and nitrogen metabolism [87]. The study underscored metabolomics as a vital tool for capturing host–environment interactions in toxicity risk, advocating its integration into precision oncology for early intervention and personalized monitoring strategies.

Doxorubicin, as the core drug of BC chemotherapy, has significantly improved the survival rate of patients, but its cardiotoxicity, such as left ventricular dysfunction and autonomic nervous disorders, is still an important challenge in clinical management [88]. A recent study by Thonusin et al. [89] investigated the role of HER2 status in shaping metabolomic profiles associated with doxorubicin-induced cardiotoxicity in BC patients. Using liquid chromatography coupled with Quadrupole time-of-flight mass spectrometry (LC/Q-TOF MS) to analyze 85 plasma metabolites, the study revealed distinct metabolic perturbations in HER2-positive versus HER2-negative patients after two weeks post-doxorubicin treatment [89]. HER2-positive patients exhibited glycine depletion, phenylalanine elevation, and reduced medium-/long-chain acylcarnitines, linked to impaired fatty acid oxidation. In contrast, HER2-negative patients showed increased glutamine, decreased tryptophan, and phosphatidylglycerol depletion, correlating with oxidative stress and autonomic dysfunction. Notably, 23 metabolites displayed consistent trends across groups [89]. However, HER2-negative patients have cardiac autonomic nervous imbalance and systemic oxidative stress, which may be caused by the loss of antioxidant protection in the HER2 pathway. In HER2-positive patients, a decrease in glycine was negatively associated with a decrease in LVEF, while a decrease in phosphatidylglycerol was positively associated with an increase in the LF/HF ratio in HER2-negative patients. These HER2-dependent metabolic signatures highlighted amino acid and lipid metabolism as key pathways in toxicity progression, with glutamine elevation in HER2-negative patients inversely correlating with later cardiac recovery [89]. The findings positioned blood metabolomes as non-invasive biomarkers for personalized risk stratification and advocate for HER2-tailored interventions, such as antioxidant supplementation or PPARα activation, to mitigate cardiotoxicity. Similarly, Lyon et al. [90] conducted the longitudinal analysis of serum metabolomic changes in 70 patients with early-stage BC before chemotherapy and one year after chemotherapy using UHPLC-HRMS techniques for non-targeted metabolomic analysis. Significant changes were found in several metabolic pathways after chemotherapy, including lysine degradation, branched-chain amino acid synthesis, linoleic acid metabolism, tyrosine metabolism and biosynthesis of unsaturated fatty acids as the top five metabolic pathways [90]. Their research elucidated previously unrecognized mechanisms that could contribute to the long-term cardiovascular toxicities associated with chemotherapy in BC survivors, highlighting the need for targeted metabolic monitoring and preventive strategies.

In addition to cardiac toxicity, a study conducted non-targeted metabolomics analysis using LC-MS on the serum samples of 50 patients with locally advanced BC before NACT, which predicted the hematological toxicity associated with NACT [91]. The study found that 53 baseline serum metabolites were significantly correlated with the lowest values of hemoglobin, white blood cells, platelets, and neutrophils after NACT. Among them, uridine, norleucine, N-acetylneuraminic acid, and 5-oxo-D-proline were listed as key metabolites, which were closely related to the decrease in platelets, neutrophils, red blood cells, and hemoglobin, respectively [91].

### 3.5. Recognizing Biomarkers for Breast Cancer Prognosis

In the study by Giskeodegard et al. [92], metabolic profiling of BC tissues using NMR revealed that elevated lactate and glycine levels correlated with reduced 5-year survival rates in ER-positive BC patients treated with primary surgery without prior NACT. This association was not detected in ER-negative cases, potentially due to the subgroup’s limited sample size and inherent metabolic heterogeneity between ER-positive and ER-negative tumor subtypes. Cao et al. [93] reported similar findings that elevated lactate levels on post-treatment tumor samples were linked to poorer prognosis, whereas decreased levels of glycine and choline-containing compounds were associated with improved outcomes. Patients who remained disease-free after five years of follow-up exhibited higher glucose levels in response to treatment compared to non-survivors.

Debik et al. [94] analyzed tissue samples from 132 women undergoing NACT and revealed that the metabolic profile of tumor biopsies detected during treatment could predict 5-year survival of patients. Patients with shorter survival had higher lactate and glycine levels in comparison with disease-free patients after five years. Increased lactate levels after treatment may reflect the activation of aerobic glycolysis and tumor response to hypoxia that led to high tumor aggressiveness and poor prognosis. Conversely, decreased glycine and tCho levels in response to treatment may be related to altered glycolysis and reduced cell proliferation, as an expression of lower disease aggressiveness and better prognosis.

Another study using NMR to analyze the relationship between dynamic changes in serum metabolism and survival prognosis before and after treatment in 80 BC patients receiving NACT. Elevated histidine and lactate levels were found to be significantly associated with improved disease-free survival (DFS), while elevated serine and taurine levels suggested an increased risk of DFS. A metabolism-related survival score (MRSS) based on these four metabolites divided patients into low-risk and high-risk groups, with a 3.42-fold and 3.34-fold increase in DFS and OS risk in high-risk groups, respectively [95].

## 4. Future Perspectives and Conclusions

Metabolomics offers a transformative perspective for exploring the dynamic progression of BC. By systematically analyzing the dynamic changes in metabolites in the tumor and tumor microenvironment, it revealed specific metabolic molecular signatures that evolve with disease stages. At the diagnostic stage, metabolomics focused on systemic alterations in lipid metabolism, such as triglycerides and phosphatidylcholines, and amino acid profiles, including phenylalanine and histidine. These changes reflected early tumor–host interactions and provide high sensitivity for early detection, which is often neglected by conventional imaging. As the disease progresses, metabolomics focuses on detecting BC recurrence and metastasis. For instance, metabolic biomarkers such as hypoxanthine and specific acylcarnitines could serve as indicators of microenvironment remodeling, EMT, and metabolic plasticity. In terms of treatment response, metabolomics revealed metabolic reprogramming related to therapeutic efficacy. For instance, elevated levels of docosahexaenoic acid (DHA) and secondary bile acids are significantly associated with pCR in neoadjuvant treatment. Upregulation of fatty acid β-oxidation (FAO) and glutamine metabolic dependence served as hallmarks of endocrine and chemotherapy resistance. The above findings highlighted the ability of metabolomics in the entire management process of BC, which will facilitate the development of precision medicine in BC.

However, almost all of the metabolite biomarkers are still in the experimental stage, and few are in the clinical application stage. A major challenge faced by metabolomics lies in the complex source and numerous confounding factors compared with other omics approaches. While metabolomics could provide comprehensive pictures of tumor patients, it highly relies on analytical methods and sample size to identify the truly relevant metabolites. For instance, the composition of plasma metabolites reflects a dynamic interplay of hepatic, muscular, and systemic organ-level metabolism, dietary influences, microbiome activity, and various other contributing factors. Cancer patients are often accompanied by other chronic diseases, so it is difficult to recognize whether changes in plasma metabolomics are due to the influence of tumor metabolism or other diseases. This poses challenges to the accuracy and specificity of metabolomic biomarkers used to improve cancer diagnosis and predict treatment responses and prognosis. Moreover, the lack of standardized protocols, extensive data repositories, affordable instrumentation, and user-friendly analysis platforms also limits the further use of metabolomics. Currently, most metabolomics studies are based on small sample retrospective cohorts and lack sufficient credibility. Prospective validation of a multicenter large population cohort is needed, which can validate the clinical utility and facilitate the true clinical translational application of metabolomics findings.

As for the future directions, only by integrating metabolomics with other omics approaches, including transcriptomics and proteomics, can we comprehensively discover functionally and diagnostically significant changes related to the different clinical stages of BC. In addition, the rapid development of artificial intelligence (AI) has promoted the application of metabolomics in cancer research, significantly improving data parsing capabilities, pathway prediction accuracy, and multi-omics integration efficiency. In terms of metabolite identification and annotation, AI-driven algorithms such as deep learning and graph neural networks can efficiently recognize the functional metabolites. Such models will enable precise molecular subtyping and more effective clinical decision-making. In the future, with the development of multiple omics, including metabolomics, and the application of AI in the medical field, it will bring new breakthroughs in medical research. Furthermore, another important direction is to develop metabolic inhibitors as therapeutic strategies. Identifying specific subtype metabolic-dependent characteristics provides new targets for treatment. For instance, inhibiting enzymes in key metabolic pathways such as lipid metabolism and glycolysis shows great potential. These targeted interventions may help bypass traditional drug resistance and improve the prognosis of patients. Finally, the clinical translation of metabolomics findings remains a priority. Future research should focus on validating metabolic signatures in large-scale, prospective multicenter cohorts, thereby promoting the clinical application of metabolomic biomarkers.

## Figures and Tables

**Table 3:** Summary of metabolomics biomarkers for breast cancer treatment response.

Sample Type	Technique Used	Changes in Metabolites	Reference
Plasma from patients who achieved pCR after NACT for BC	LC-HRMS	docosahexaenoic acid (↑), preoperative glycine deoxycholic acid (↑), glycine hyodeoxycholic acid (↑)	[71]
Plasma from patients who achieved pCR after NACT for BC	CE-MS, LC-MS	ADP (↓), hydroxyproline (↓), N-acetylaspartate (↑), proline (↑), 3-Indoxyl sulfate (↓), creatine (↓) and uric acid (↓), 4-methyl-2-oxo-valerate (↓) and uric acid (↓), asparagine (↑)	[72]
Plasma from patients who achieved pCR after NACT for BC	LC-MS, GC-MS	sophorobiose (↑), n-(2-acetamido) iminodiacetic acid (↑), taurine (↓), 6-hydroxy-2-aminocaproic acid (↑)	[73]
Plasma from patients who achieved pCR after NACT for BC	LC-MS	spermidine (↑), tryptophan (↓)	[74]
Plasma from patients who achieved pCR after NACT for BC	NMR, LC-MS	threonine (↓), glutamine (↓), isoleucine (↑), histidine, linolenic acid (↓)	[75]

↑—increase, ↓—decrease.

## Data Availability

No new data were created or analyzed in this study. Data sharing is not applicable to this article.
